# A Scheduling Method of Using Multiple SAR Satellites to Observe a Large Area

**DOI:** 10.3390/s23063353

**Published:** 2023-03-22

**Authors:** Qicun Zheng, Haixia Yue, Dacheng Liu, Xiaoxue Jia

**Affiliations:** 1 Department of Space Microwave Remote Sensing System, Aerospace Information Research Institute, Chinese Academy of Sciences, Beijing 100190, China; 2 School of Electronic, Electrical and Communication Engineering, University of Chinese Academy of Sciences, Beijing 100049, China

**Keywords:** multiple SAR satellites, scheduling method, grid space, strip generation, variable neighborhood tabu search algorithm

## Abstract

This paper presents a scheduling problem of using multiple synthetic aperture radar (SAR) satellites to observe a large irregular area (SMA). SMA is usually considered as a kind of nonlinear combinatorial optimized problem and its solution space strongly coupled with geometry grows exponentially with the increasing magnitude of SMA. It is assumed that each solution of SMA yields a profit associated with the acquired portion of the target area, and the objective of this paper is to find the optimal solution yielding the maximal profit. The SMA is solved by means of a new method composed of three successive phases, namely, grid space construction, candidate strip generation and strip selection. First, the grid space construction is proposed to discretize the irregular area into a set of points in a specific plane rectangular coordinate system and calculate the total profit of a solution of SMA. Then, the candidate strip generation is designed to produce numerous candidate strips based on the grid space of the first phase. At last, in the strip selection, the optimal schedule for all the SAR satellites is developed based on the result of the candidate strip generation. In addition, this paper proposes a normalized grid space construction algorithm, a candidate strip generation algorithm and a tabu search algorithm with variable neighborhoods for the three successive phases, respectively. To verify the effectiveness of the proposed method in this paper, we perform simulation experiments on several scenarios and compare our method with the other seven methods. Compared to the best of the other seven methods, our proposed method can improve profit by 6.38% using the same resources.

## 1. Introduction

Synthetic aperture radar (SAR) satellites can acquire images of specified areas of the Earth’s surface at observation requests with all-day and all-weather imaging capacity [1,2]. Image products acquired by SAR satellites are widely used in many fields, such as emergency response in environments [3], target recognition and classification [4], urban planning and architectural layout [5], and crop detection and disaster prevention [6,7]. With the enhancement of imaging capabilities of SAR and the rapid growth in the number of SAR satellites, customers are increasingly demanding timeliness in SAR image acquisition [8]. Therefore, it is necessary to use multiple SAR satellites with similar spatial resolutions for ensuring quicker access to images of the target area [9].

The SMA can be described as follows: There is a large irregular area to be observed and several SAR satellites on various orbits. The aim is to develop the optimal observation schedule for each SAR satellite to acquire the maximal profit during the given schedule time horizon. Therefore, SMA is a non-deterministic polynomial (NP-Hard) [10] and is highly relevant to inventory [11], allocation [12], backpacking [13] and machine scheduling problems [14].

The difficulties in solving the SMA are as follows: (1) The SMA, which is strongly coupled with geometry, has complicated forms and many constraints. (2) The variable that represents the look angle takes its value from a continuous interval. Hence, the number of solutions is infinite. (2) Taking the overlap between strips and the irregularity of the target area into account, it is difficult to directly calculate the coverage of a specific strip. Based on the above aspects, previous studies adopt heuristic algorithms and their variants instead of exact algorithms to find the optimal solution of the SMA.

The current method to solve the SMA is composed of three successive phases, namely, grid space construction, target decomposing and strip selection (GTS) [15]. In the grid space construction, an area discretization method is proposed to discretize the target area into a set of points in a specific plane rectangular coordinate system and calculate the total profit of a solution, which is a subset of candidate strips. In the target decomposing, the target area is split into several parallel candidate strips following the direction of the subpoint trajectory of the SAR satellite. The SAR satellite could capture the whole image of the region corresponding to a candidate strip in a single orbit during the given schedule time horizon. The way of parallel splitting allows the variable that represents the look angle to take values from discrete space, thus reducing the complexity of the SMA [15]. In the strip selection, a solution is a subset of candidate strips and the aim is to find the optimal solution corresponding to the maximum profit in solution space. 

The GTS can be further improved to solve the SMA. In the grid space construction, it is easy to generate a grid space in a rectangular area, whereas the target area to be observed in the imaging mission is generally irregular. Therefore, designing a normalized strategy to generate a grid space for an irregular area is necessary. In the target decomposing, the parallel split method produces several parallel candidate strips with a fixed length and location at each orbit of the SAR satellite. Hence, some better solutions in a global context are likely to be excluded in the strip selection. An effective alternative strategy is to generate more candidate strips with flexible positions and lengths according to the grid of the first phase. Thus, there will be more high-quality solutions available for the strip selection phase. Accordingly, a new three-phase method for SMA is proposed in this paper and the main improvements are made as follows.

(1) A new three-phase method composed of grid space construction, candidate strip generation and strip selection (GCS) is developed to solve the SMA in this paper. The main difference between GCS and GTS is the second phase in which grid split is applied in GCS whereas parallel split is applied in GTS. Compared with parallel split strategy, grid split strategy could produce more candidate strips with a flexible position and length. Hence, there will be more high-quality solutions available for the strip selection phase. (2) This paper develops a normalized grid space generation algorithm, a candidate strip generation algorithm and a variable neighborhood tabu search algorithm (VNTS) in the three successive phases of the GCS. (3) To verify the advantages of the GCS–VNTS method in this paper, we perform several numerical tests and compare the other seven solution methods. The simulation results indicate that the GCS–VNTS method proposed in this paper outperforms the other methods with the same resource consumption.

At present, there is little research on SMA. The SMA is a variant of the polygon geometric coverage problem [16], which is essentially an NP-Hard combinatorial optimization problem [10]. The polygonal geometry coverage problem and satellite mission scheduling problem are highly related to SMA. In the field of polygonal geometry coverage, the square coverage optimization problem is one of the classical problems and usually uses multiple small squares to cover a big rectangle. Study [17] studied the optimization problem of orthogonally covering a big square with some small unequal squares and tried to find the largest side length of the big square, which is similar to the situation of acquiring a larger coverage with limited resources. In contrast to study [17], study [18] discussed the relation between the number of unit squares and the side length of the big square, deriving the upper and lower bound on the number of unit squares. Study [19] further investigated the issue and recomputed the lower bound when n=2 or n=3. In addition, the problem of how to use the fewest unit squares to cover some given line segments is interesting; study [20] proved that it is an NP-Hard problem in some cases. For the first time, study [21] put a polynomial time approximation algorithm forward, using axis-parallel rectangles to cover a rectilinear polygon with holes. 

Among the many studies in the field of polygonal geometry coverage, study [22] proposed the Single Frame Selection (SFS) problem with satellite imaging as the background and designed an algorithm with polynomial time complexity. The SFS problem is to find the best location of a single frame, yielding the maximal reward during each time window. In addition, study [23] designed an approximation algorithm and an improved branch-and-bound algorithm based on the SFS problem, analyzing the tradeoff between solution quality and the corresponding computation time. By further studying the SFS problem, study [24] generalized the SFS problem for a single imaging opportunity to the case of multiple imaging opportunities, proposed the Multiple Frame Selection (MFS) problem and developed a corresponding greedy algorithm. The above three articles proposed the SFS and MFS problems and applied several efficient algorithms. However, their optimization problems are aimed at the same scanning direction of a satellite and do not consider the difference in imaging direction and size during cooperative scanning of multiple satellites. In addition, the optimized objective functions are also different.

The satellite mission scheduling problem is one of the classical computational optimization problems, which is composed of the target point and target area mission scheduling problem. Study [25] first proposed a test data set of the target point scheduling problem for SPOT satellites. Studies [26,27] presented a formulation of the problem that includes numerous binary and ternary logical constraints, and they tested the tabu search algorithm with realistic benchmark instances. To solve the satellite mission scheduling problem, study [28] proved that one of the several 0–1 linear programs presents a smaller integrality gap. In addition, there were several mathematical programming methods, such as derived algorithms using graph theory concepts [29], enumeration and interactive selection algorithm of a multiple criteria path in a graph without circuit [30], and column generation algorithm with decomposition techniques [31]. Mathematical programming algorithms may find optimal solutions, but generally consume enormous resources [29,30,31]. Studies [32,33] presented a two-phase scheduling method for the satellite scheduling problem. To solve the real-time scheduling problem of Earth observation satellites, study [34] established a scheduling model with multiple objectives and presented a new real-time processing algorithm. 

In the field of satellite mission scheduling for a target area, study [35] proposed four methods, including a local search algorithm, constraint programming algorithm, dynamic programming algorithm and heuristic algorithm by using the parallel segmentation strategy. Studies [36,37,38] presented a tabu search heuristic algorithm to select a subset of requests to maximize profit. Studies [39,40] used the biased random key genetic and local search heuristic algorithm to solve a multi-user observation scheduling problem. Study [41] considered many technical and managerial constraints and developed a constructive algorithm that produced a feasible plan in a very short time. Study [42] proposed a heuristic algorithm to develop the schedules and depicted the components of a decision support system for environmental monitoring satellites. The above authors made considerable achievements in the field of polygonal geometry coverage and satellite mission scheduling, but their mathematical models and algorithms could not directly be applied to solve the SMA.

The objective of this paper is to find the optimal solution of the SMA, yielding the maximal profit, and the main improvements in this paper are made as follows.

(1) A new three-phase GCS method, which is composed of grid space construction, candidate strip generation and strip selection, is developed to solve the SMA in this paper. Compared to the GTS method, the GCS method could produce more candidate strips with a flexible position and length in the second phase. Therefore, there will be more high-quality solutions available for the third phase of strip selection. (2) This paper develops a normalized grid space generation algorithm, a candidate strip generation algorithm and a VNTS algorithm in the three successive phases of GCS. (3) To verify the advantages of the GCS–VNTS method in this paper, we perform several numerical tests and compare the other seven methods. The simulation results indicate that the GCS–VNTS method proposed in this paper outperforms the other seven methods with the same resource consumption. 

The remainder of this paper is organized as follows: we introduce the three phases of GCS and the corresponding three algorithms in detail in Section 2, perform simulation experiments on several scenarios and discuss simulation results in Section 3, and give the conclusion in Section 4.

## 2. Materials and Methods

### 2.1. Problem Description

The target area waiting to be observed is a large region of the Earth’s surface and a finite number of SAR satellites on various orbits are available to perform imaging operations. The size of the target area is much wider than the swath width of a SAR. Hence, the target area cannot be photographed by a SAR satellite in a single shot. Typically, only part of the images of the target area could be captured by SAR satellites during the given schedule time horizon because the SMA is usually an over-subscribed problem. Each solution of SMA yields a total profit associated with the acquired portion of the target area and the value of profit is a normalized number within the range of [0, 1]. The objective is to find the optimal solution yielding the maximal profit. To better address the SMA, several terms are defined as follows:

- Observation opportunity:

The observation window when a SAR satellite passes over the target area. Generally, there are multiple observation opportunities for a SAR satellite to observe the target area during the given schedule time horizon.

- Observation pattern:

The pattern formed by setting the observation start time, the observation end time and the look angle of an observation opportunity. Each observation pattern corresponds to a candidate strip of fixed swath width and variable length proportional to the observation duration.

- Schedule:

Observation scheme formed by selecting a candidate strip, namely, the observation pattern for each observation opportunity. Each observation schedule corresponds to a solution of SMA.

**Figure 1:**
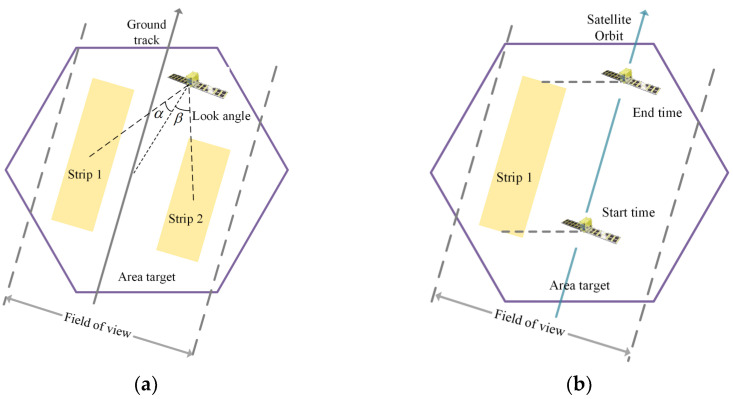
Schematic illustration of SAR satellite imaging. (**a**) Strips with different look angles; (**b**) start time and end time of a strip.

The images of the target area can be only captured within the field of view (FOV) of a SAR satellite in an observation opportunity. FOV refers to the spatial scope that the SAR satellite can observe in an observation opportunity. Typically, FOV is much larger than a strip, which indicates that an observation opportunity could flexibly select the optimal one from multiple candidate strips, as presented in Figure 1.

**Figure 2:**
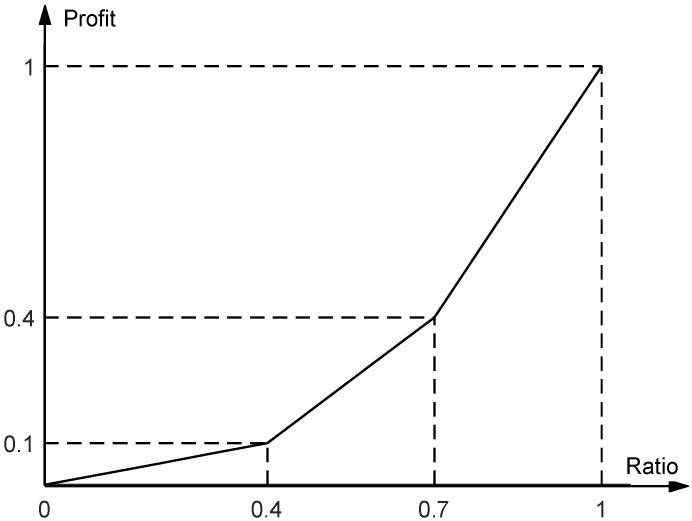
The piecewise linear function for profit calculation.

The SMA in this paper refers to multiple SAR satellites on multiple orbits and multiple observation opportunities. Moreover, there are multiple observation patterns, namely, multiple candidate strips in an observation opportunity. The schedule for all the SAR satellites is a subset of candidate strips. Each schedule generates a different profit. The profit associated with the acquired portion of the target area is defined by means of a piecewise linear convex function [36], which takes values from the range [0, 1], as presented in Figure 2. It should be noted that the value of profit will become higher as the imaged area ratio (ratio of the acreage of the imaged area to the acreage of the whole target area) increases and the value of profit will be 1 if the target area is completely imaged. This also indicates that partial images have little value to customers and we focus on the total profit to evaluate a schedule. 

### 2.2. Problem Formulation

#### 2.2.1. Assumptions and Simplifications

Since the SMA in this paper is NP-Hard [10], it is necessary to simplify the SAR satellite scheduling process before modeling. The simplifications we have made are as follows:

1. All the SAR satellites will only perform this mission during the given schedule time horizon and there is only one SAR running on a SAR satellite;
2. Operation mode of the SAR is the broadside strip;
3. Only one observation pattern, namely, one candidate strip, is selected for each observation opportunity;
4. No consideration is given to the data download process of the SAR images captured by satellites;
5. Resolution variation in the SAR satellites during one mission is considered acceptable in a certain range.

#### 2.2.2. Sets and Parameters

The mathematical formulation is developed to solve the SMA and the notations used in the formulation are presented as follows.

- R, the imaging request for the specified target area from the customer. The attributes of R are defined as follows:
  *A* is the target area to be imaged.
  *B* is the required start time of R.
  *E* is the required end time of R.
- $S=\left\{s_{1},s_{2},\ldots ,s_{\left|S\right|}\right\}$, the set of SAR satellites. The attributes of $s_{i}$ are defined as follows:
  $T_{i}$ is the maximum imaging duration of a single orbit of $s_{i}$.
  $G_{i}$ is the maximum look angle of $s_{i}$.
- $O_{i}=\left\{o_{i1},o_{i2},\ldots ,o_{i\left|O_{i}\right|}\right\}$ is the set of observation opportunities of $s_{i}$ during the given schedule time horizon. For $o_{ij}$, the following attributes are defined:
  $s_{ij}$ is the calculated start time of $o_{ij}$.
  $e_{ij}$ is the calculated end time of $o_{ij}$.
- $P_{ij}=\left\{p_{ij1},p_{ij2},\ldots ,p_{ij\left|P_{ij}\right|},\right\}$ is the set of observation patterns of $o_{ij}$. For $p_{ijk}$, the following attributes are defined:
  $b_{ijk}$ is the calculated start time of $p_{ijk}$.
  $c_{ijk}$ is the calculated end time of $p_{ijk}$.
  $g_{ijk}$ is the maximum look angle of $p_{ijk}$.
- Two functions, f and C, involved in the mathematical formulation are defined as follows:

f is the piecewise linear convex function formed by four points, namely, (0,0), (0.4,0.1), (0.7,0.4) and (1,1). The function f is proposed to describe the relationship between total profit and coverage ratio of a schedule.

C is the area calculation function involved in quantifying the rate of coverage for schedules.

- Decision variable:

(1) $$x_{ijk}=\left\{\begin{array}{c} 1,\mathrm{the}p_{ijk}\mathrm{is}\mathrm{selected}\mathrm{in}\mathrm{schedule}\\ 0,\mathrm{otherwise} \end{array}\right.$$

#### 2.2.3. Mathematical Formulation

Based on the above problem simplification and parameter setting, the mathematical formulation of the SMA can be illustrated as follows:

(2) $$\max f\left[\frac{C\left(\cup_{i=1}^{\left|S\right|}\cup_{j=1}^{\left|O_{i}\right|}\cup_{k=1}^{\left|P_{ij}\right|}x_{ijk}*p_{ijk}\right)}{C\left(A\right)}\right]$$

(3) $$\overset{\left|P_{ij}\right|}{\underset{k=1}{\sum }}x_{ijk}\leq 1,\forall i\in \left[1,\left|S\right|\right],\forall j\in \left[1,\left|O_{i}\right|\right]$$

(4) $$B\leq s_{ij}\leq e_{ij}\leq E,\forall i\in \left[1,\left|S\right|\right],\forall j\in \left[1,\left|O_{i}\right|\right]$$

(5) $$s_{ij}\leq b_{ijk}\leq c_{ijk}\leq e_{ij},\forall i\in \left[1,\left|S\right|\right],\forall j\in \left[1,\left|O_{i}\right|\right],\forall k\in \left[1,\left|P_{ij}\right|\right]$$

(6) $$c_{ijk}-b_{ijk}\leq T_{i},\forall i\in \left[1,\left|S\right|\right],\forall j\in \left[1,\left|O_{i}\right|\right],\forall k\in \left[1,\left|P_{ij}\right|\right]$$

(7) $$g_{ijk}\leq G_{i},\forall i\in \left[1,\left|S\right|\right],\forall j\in \left[1,\left|O_{i}\right|\right],\forall k\in \left[1,\left|P_{ij}\right|\right]$$

Objective function (2) is intended to maximize the total profit of the observation schedule. The total profit determines the quality of the observation schedule.

Constraint (3) indicates that, at most, one observation pattern is selected for each observation opportunity. 

Constraints (4) and (5) ensure that all observation operations must be executed within the given schedule time horizon. 

Constraint (6) indicates that each candidate strip has a limitation of length proportional to the maximum imaging duration of a single orbit of a SAR satellite, subject to the constraint of energy capacity of the SAR satellite. 

Constraint (7) is an illustration of the fact that the limitation of the maneuvering ability of the SAR satellite cannot be ignored.

### 2.3. Three-Phase Method (GCS)

**Figure 3:**
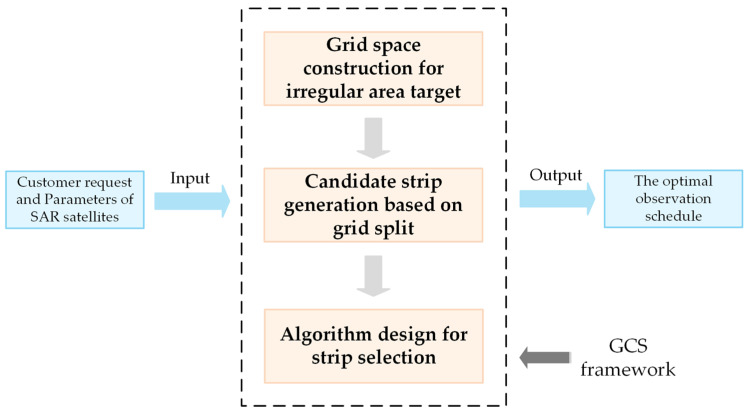
Main GCS framework for SMA.

To solve the SMA, a new GCS method composed of grid space construction, candidate strip generation and strip selection is proposed in this paper. The grid space construction is developed to discretize the irregular area into a set of points in a specific plane rectangular coordinate system and calculate the total profit of a solution of SMA. Then, the candidate strip generation is designed to produce numerous candidate strips based on grid space. The traditional parallel split method produces several parallel candidate strips with a fixed length and location at each orbit of each SAR satellite [15]. Therefore, the size of solution space is limited. The candidate strip generation method based on grid split could produce more candidate strips with flexible positions and lengths. Thus, there will be more high-quality solutions available for the strip selection phase. At last, in the strip selection process, each subset of candidate strips corresponds to a solution and the total profit determines the quality of a solution. A specific heuristic algorithm is designed to search the optimal solution from the whole solution space. The main framework of the GCS is depicted in Figure 3 and the detailed modules of the GCS are as follows.

#### 2.3.1. Grid Space Construction

As the Earth is an irregular ellipsoid and the target area is a large irregular area, the region covered by a strip on the ground is not a standard rectangle. To acquire the obtained profit of a strip, the target area is always discretized by a grid with a specific step, reducing the couple with computational geometry [15]. Then, observing all cells in a grid of the target area represents approximately observing the whole target area. Based on that, a point is placed in the middle of a cell. If a strip covers a point, the area of the cell corresponding to that point is completely observed. The number of points covered by a strip is summed to calculate the profit of the strip. As described above, the target area is a large irregular area. Therefore, it is necessary to develop a normalized grid generation algorithm. In this paper, the surface of the target area is gridded in a specific plane rectangular coordinate system. 

**Figure 4:**
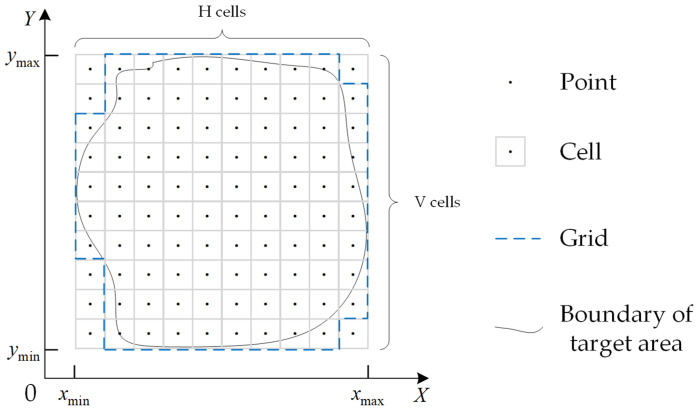
Schematic diagram of the grid space construction.

First, a rectangular area is defined according to the minimum horizontal coordinate $x_{\min}$, maximum horizontal coordinate $x_{\max}$, minimum vertical coordinate $y_{\min}$ and maximum vertical coordinate $x_{\max}$ of the boundary vertices of the target area. Then, the rectangular area is divided into a set of successive square cells with a specific step. As depicted in Figure 4, the set of these cells following the original spatial relations is called a grid, denoted as follows:

(8) $$E=\overset{H}{\underset{\alpha =1}{\cup }}\overset{V}{\underset{\beta =1}{\cup }}\left\{e_{\alpha ,\beta }\right\}$$

$e_{\alpha ,\beta }$ denotes the cell whose upper right X–Y coordinates are presented as follows:

(9) $$x=x_{\min}+\frac{\alpha \left(x_{\max}-x_{\min}\right)}{H}$$

(10) $$y=y_{\min}+\frac{\beta \left(y_{\max}-y_{\min}\right)}{V}$$

It should be noticed that only a grid formed by the blue dotted line in Figure 4 needs to be covered.

#### 2.3.2. Candidate Strip Generation

The main existing method for generating a candidate strip is the parallel split method [15]. In the target decomposing phase, the parallel split method produces several parallel strips with fixed lengths and locations and simplifies the SMA because the look angle takes values from discrete space. However, the parallel split method only considers the look angle and ignores the start and end times of the candidate strip, resulting in a limited number and flexibility of candidate strips. Hence, some better solutions in a global context are likely to be excluded in the strip selection.

**Figure 5:**
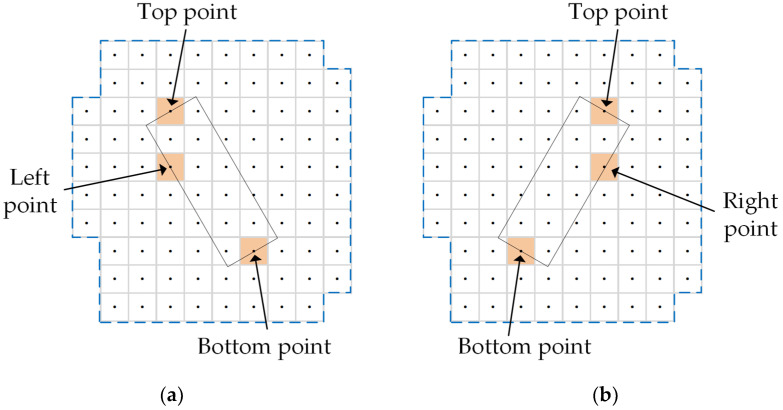
Candidate strip based on grid. (**a**) Ascending; (**b**) descending.

An efficient alternative strategy is to generate more strips with flexible positions and lengths according to the grid of the first phase. Thus, there will be more high-quality solutions available for the strip selection. Accordingly, this paper develops a new candidate strip generation algorithm with respect to three degrees of freedom, namely, look angle, start time and end time. It is obvious that a candidate strip needs three points to determine its position and length. The bottom point and top point determine the imaging start time and imaging end time of the observation pattern, respectively, whereas the left point or right point determines the look angle of the imaging pattern, as depicted in Figure 5. It should be noticed that only three points that respect all constraints can form a valid candidate strip. Once a candidate strip is chosen to perform, all points covered by this strip are obtained and the three points forming this strip are also considered to be covered by this strip. 

**Figure 6:**
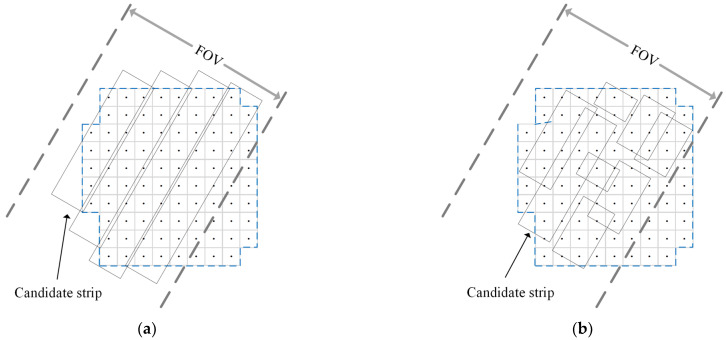
Two modes to generate candidate strip. (**a**) Parallel split; (**b**) grid split proposed in this paper.

Compared with the parallel spilt method, the way of the grid split has higher degrees of freedom and can produce more candidate strips with a flexible position and length, as depicted in Figure 6. The steps of the candidate strip generation algorithm based on the grid split are shown in Algorithm 1.
**Algorithm 1** Candidate strip generation algorithm.**Input:** Imaging opportunity z and the set M of points. **Output:** The set η of candidate strips. for $i\leftarrow 1;i\leq \left|M\right|;i++$  for $j\leftarrow 1;j\leq \left|M\right|;j++$   for $k\leftarrow 1;k\leq \left|M\right|;k++$    if three points $\langle M\left(i\right),M\left(j\right),M\left(k\right)\rangle$ meet constraints of z     generate candidate strip ρ according to $\langle M\left(i\right),M\left(j\right),M\left(k\right)\rangle$ and z;     then $\eta \leftarrow \left\{\eta ,\rho \right\};$    end   end  end end

#### 2.3.3. Strip Selection Phase

**Figure 7:**
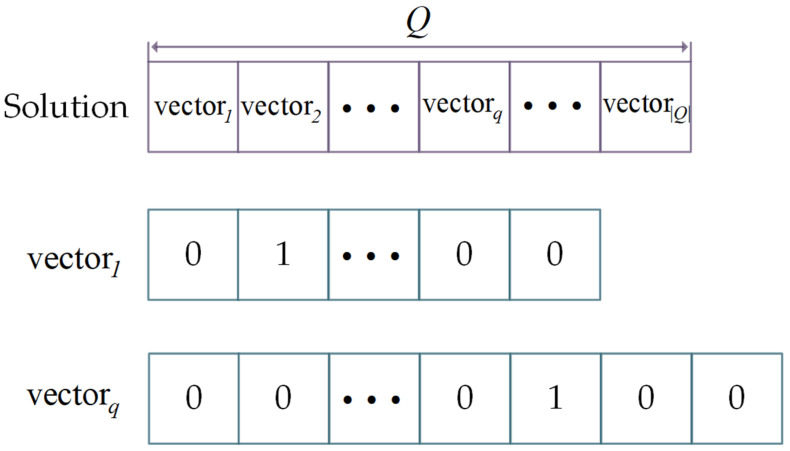
0–1 Integer vector coding method.

In strip selection, for each imaging opportunity, only one strip is selected to perform from all the candidate strips. The objective of strip selection is to find the optimal subset of candidate strips yielding the maximal profit. Based on that, the criterion for selecting strips is to make the number of points covered by selected strips as large as possible. Therefore, strip selection is a kind of set-covering problem [43], which is NP-Hard in a strong sense. A solution of the strip selection comprises a number of 0–1 integer vectors equal to the number of the observation opportunities Q in the given schedule time horizon, as illustrated in Figure 7.

(11) $$Q=\overset{\left|S\right|}{\underset{i=1}{\sum }}\left|O_{i}\right|$$

Each 0–1 integer vector corresponds to an observation opportunity and the length of 0–1 integer vector equals the number of observation patterns. A value of 1 represents that the observation pattern is chosen, whereas a value of 0 indicates that the observation pattern is not scheduled. 

As the strip selection is NP-Hard in a strong sense, it is difficult to directly find the optimal subset of candidate strips using exact algorithms. However, heuristic algorithms and its variants, such as the genetic algorithm (GA) [15], tabu search algorithm (TS) [36] and simulated annealing algorithm (SA) [44], are effective for solving the SMA. This paper further improves the neighborhood structure of TS and proposes a variable neighborhood tabu search algorithm (VNTS) to search the optimal solution. There are two neighborhoods, namely, the base neighborhood and extended neighborhood developed in the VNTS. In the searching process of the VNTS, the basic neighborhood is designed to quickly find the local optimal solution of the current subspace, whereas the extended neighborhood is designed to enter a new subspace by expanding the scope of the current space. Alternating two neighborhoods can effectively improve the search efficiency and prevent the searching process from being trapped in a local subspace. At each iteration of the search process, VNTS explores the solution space of SMA by moving from the current solution s to the “best” solution in its neighborhood $N\left(s\right)$.

- Base neighborhood:

As described above, the solution is a subset of candidate strips. Therefore, selecting an observation opportunity based on the current solution and iterating through all the observation patterns of this observation opportunity, excepting the current observation pattern, can generate a number of neighborhood solutions. The base neighborhood is composed of the set of neighborhood solutions generated by selecting multiple observation opportunities in the above manner. If the number of observation patterns for each selected observation opportunity is α and the number of selected observation opportunities is β, the summed number of solutions of base neighborhood is $\left(\alpha -1\right)\beta$.

- Extended neighborhood:

Selecting a pair of observation opportunities and simultaneously combining its observation patterns can generate a number of neighborhood solutions. The extended neighborhood is composed of the set of neighborhood solutions generated by selecting several pairs of observation opportunities in the above manner. If the number of observation patterns for each selected observation opportunity is α and the number of selected observation opportunities is β, the summed number of solutions of extended neighborhood is $\left(\alpha^{2}-1\right)\beta /2$. 

**Figure 8:**
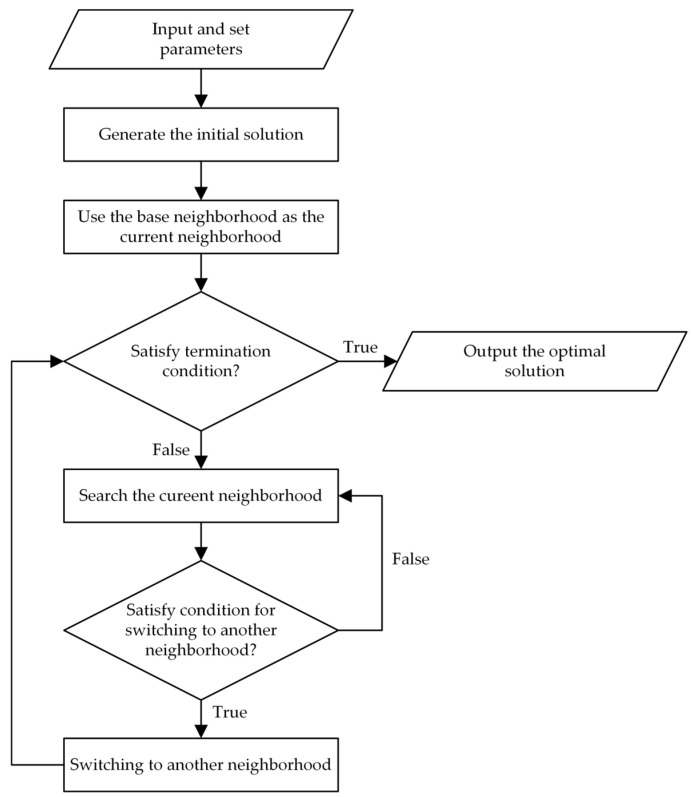
The flow diagram of the VNTS proposed in strip selection.

It is obvious that the scope of the search space of the extended neighborhood is much larger than that of base neighborhood. The search process is dominated in the base neighborhood and supplemented by the extended neighborhood. After completing a given number of iterations in the base neighborhood, the search process will switch to the extended neighborhood. Alternating two neighborhood structures essentially drives the algorithm to continuously search different valid subspaces. The flow diagram of the VNTS proposed in the strip selection is depicted in Figure 8 and the detailed steps of VNTS are given in Algorithm 2.
**Algorithm 2** Steps of variable neighborhood tabu search algorithm.**Input:** Candidate strips and imaging opportunities. **Output:** The feasible observation solution yielding the maximal profit. Set tabu length, termination condition and neighborhood switching condition; Generate initial solution s; Use s as the current solution and current optimal solution; Use the base neighborhood as the current neighborhood structure; **while** not termination-condition **do**  Generate the current neighborhood $N\left(s\right)$ of current solution s;  Select a new solution from $N\left(s\right)$ according to Metropolis-criterion;  **if** switching condition for another neighborhood structure **then**    Switching to another neighborhood structure; **  end if**  updating the tabu list, current solution and current optimal solution. **end while**
**return** current optimal solution; 

## 3. Results and Discussion

In this section, we performed several simulated experiments to test the effectiveness of the proposed GCS method and VNTS algorithm in various scheduling scenarios based on the Chinese SAR satellite platform. The simulation experiment was executed on a PC with Intel© (TM) i7-11700 (2.50 GHz CPU speed) and 52 GB RAM. The configurations of the scheduling scenarios are depicted as follows.

### 3.1. Simulation Parameters

#### 3.1.1. Imaging SAR Satellites

From the Chinese SAR satellite database, we chose five SAR satellites on various orbits to perform simulated experiments. They were L-SAR 01A, L-SAR 01B, GAOFEN 3, GAOFEN 3-02 and GAOFEN 3-03, whose orbit information could be acquired from the website https://celestrak.com/ (accessed on 2 November 2022). Several parameters of the five SAR satellites and their sensors are listed in Table 1.

#### 3.1.2. Imaging Areas

To verify the effectiveness of the GCS method and VNTS algorithm proposed in this paper, Gabon and Belarus, having different sizes, shapes and latitudes, were selected as the two imaging areas. The parameters of the two imaging areas are listed in Table 2.

#### 3.1.3. Imaging Time

We defined the UTCG time range from “1 January 2022 00:00:00.000” to “2 January 2022 00:00:00.000” as the given schedule time horizon to perform all the simulated experiments. The real-time spatial position of SAR satellites can be obtained based on orbit information. Then, the visible time windows of imaging opportunities can be calculated according to the relative spatial position between the five SAR satellites and the two imaging areas. The number of imaging opportunities of each SAR satellite for Belarus and Gabon is given in Table 3.

### 3.2. Comparison Results with Other Methods 

**Figure 9:**
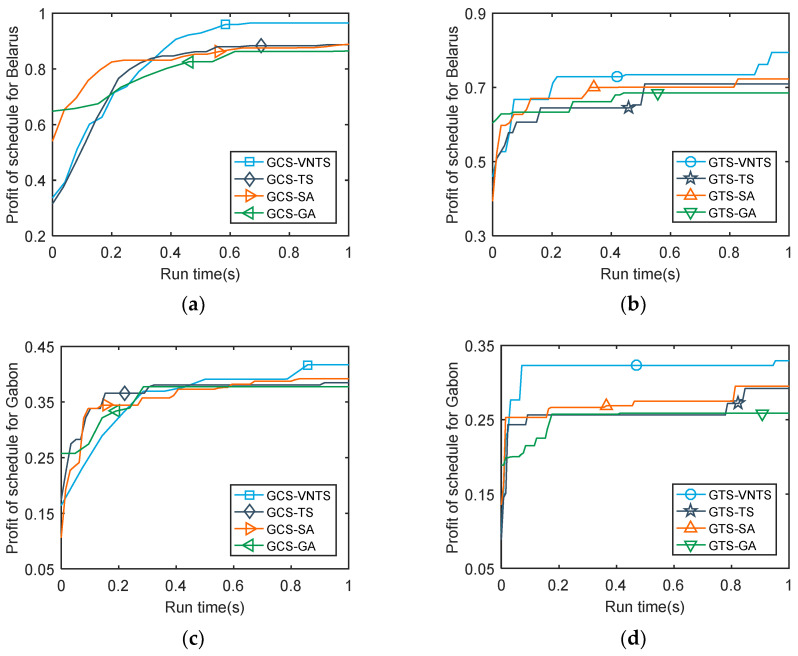
The profit of the obtained schedule versus the run time of CPU. (**a**) The profit of schedules for Belarus using the GCS strategy; (**b**) the profit of schedules for Belarus using the GTS strategy; (**c**) the profit of schedules for Gabon using the GCS strategy; (**d**) the profit of schedules for Gabon using the GTS strategy.

**Figure 10:**
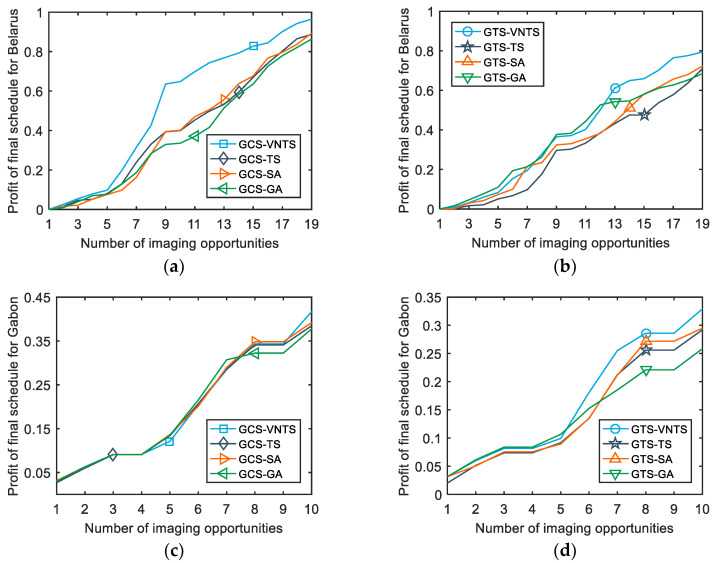
The profit of the final schedule versus the number of imaging opportunities. (**a**) The profit of the final schedule for Belarus using the GCS strategy; (**b**) the profit of the final schedule for Belarus using the GTS strategy; (**c**) the profit of the final schedule for Gabon using the GCS strategy; (**d**) the profit of the final schedule for Gabon using the GTS strategy.

There were several simulated experiments conducted to evaluate the performance of the GTS and GCS. In addition, to verify the effectiveness of the VNTS algorithm proposed in this paper, we compared the results of TS [36], SA [44], GA [15] and VNTS. Table 4 provides the profit results of the four algorithms implemented under GCS and GTS strategies, respectively, when the running time of algorithms is one second. To comprehensively compare the profit results of all the scenarios in the iteration process, we plotted the profit values of the obtained schedules versus the run time of the CPU in Figure 9. Moreover, Figure 10 shows the profit of the final schedules as the number of imaging opportunities increases. 

Table 4 provides the obtained profit results of the four algorithms implemented under the GCS and GTS strategies, respectively, when the running time of the algorithms is one second. The profit results of Table 4 show that the effect of the GCS strategy is much better than that of the GTS strategy when using the same algorithm in the third phase. When performing the imaging mission on Belarus, our GCS–VNTS method outperforms the GTS–VNTS method by 21.46% and the GCS–SA method outperforms the GTS–SA method by 25.92%. The results of the comparison of the 16 scenarios in Table 4 show that the GCS strategy has a profit improvement of more than 20% over the GTS strategy when the same algorithm is employed. In addition, the worst profit results provided by GCS–GA are better than the best results provided by GTS–VNTS. In comparison with the GTS–VNTS method, the GCS–GA method increases the profit of Belarus and Gabon by 8.80% and 14.61%, respectively. Hence, the GCS strategy is more effective than the GTS strategy when solving the SMA. The main difference between the GTS and GCS is the second phase in which the parallel split method is employed in GTS, whereas the grid split method is employed in GCS. Compared with the parallel split method, the grid split method can produce more candidate strips with flexible positions and lengths according to the grid of the first phase. Based on that, the magnitude of the solution space is increased and there will be more high-quality solutions available for the third phase.

Figure 9 shows the curves of the profit values of the obtained schedules versus the run time of the CPU in one second. The curves of the GCS show a more smoothly increasing trend versus the run time than the curves of the GTS, which indicates that the solution space of GCS is much larger than GTS and better solutions of GCS are included in the third phase of strip selection. In addition, the results of the initial solutions of GA are better than three other algorithms because the optimal one among multiple initial solutions is employed in GA, whereas a random initial solution is employed in three other algorithms. However, GA generally provides the worst values in the final schedule. In contrast to GA, VNTS searches the solution space with an ordinary random initial solution and finally obtains the best values. In addition, TS and SA generally provide similar values of the final schedules. The profit results of the final schedules for Belarus using the GCS strategy show that the VNTS gained 8.70% against the TS and 7.96% against the SA. Compared to the best of the other seven methods, our proposed GCS–VNTS method can improve profit by 6.38% using the same resources. Based on the above discussion, the GCS strategy outperforms GTS strategy by a wide margin and the proposed GCS–VNTS method outperforms the other seven methods with the same computational time consumption.

Additionally, in order to explore the details of the obtained final schedules, Figure 10 plots the profit values of the final schedules versus the number of imaging opportunities. It should be noted that the observation schedule should be re-developed in a global perspective, rather than simply adding or subtracting several strips from the original schedule when the number of observation opportunities changes. In addition, we are of the opinion that observation profit is a better indicator of value creation than the imaged area ratio. Hence, the functional relationship between the observation profit and the imaged area ratio will also affect the profit results to some extent. However, it should be noted that the change in the functional relationship between the observation profit and the imaged area ratio does not affect the effectiveness of the GCS–VNTS method proposed in this paper.

## 4. Conclusions

This paper presented the SMA problem of using multiple SAR satellites to observe a large irregular target area. We discussed the main difficulties in solving the SMA and introduced the current GCS framework. SMA is usually considered as a kind of NP-Hard combinatorial optimization problem strongly coupled with geometry. Hence, it is difficult to solve the SMA in a straightforward manner. We proposed a new GCS method composed of three successive phases, namely, grid space construction, candidate strip generation and strip selection. The grid space construction establishes the profit evaluation system and the candidate strip generation produces more flexible candidate strips based on grid split than the traditional parallel split method. At last, a VNTS algorithm was developed in the third phase to search the optimal solution yielding the maximal profit. To verify the effectiveness of the GCS–VNTS method, we performed numerical tests on several simulated instances. The computational results of the simulation experiments indicated that the GCS strategy has a profit improvement of more than 20% over the GTS strategy when the same algorithm is employed. Compared to the best of the other seven methods, our proposed GCS–VNTS method can improve profit by 6.38% using the same resources. Hence, the effectiveness of the GCS–VNTS method proposed in this paper is verified. 

## Figures and Tables

**Table 1:** Several parameters of the five selected SAR satellites and their sensors.

Name	L-SAR (01A,01B)	GAOFEN (3,3-02,3-03)
Orbit height (km)	607	755
Inclination (°)	97.83	98.43
Range of incidence angle (°)	10~60	19~50
Resolution (m)	3	5
Maximum imaging duration of a single orbit (min)	7.5	10
Swath width (km)	50	50

**Table 2:** Parameters of Belarus and Gabon.

Name	Belarus	Gabon
Maximum longitude (°)	32.74	15.53
Minimum longitude (°)	23.16	8.70
Maximum latitude (°)	56.17	2.33
Minimum latitude (°)	51.24	−3.93
Area(km^2^)	207,721	260,547

**Table 3:** Number of observation opportunities.

Name	Belarus	Gabon
L-SAR 01A	4	2
L-SAR 01B	4	2
GAOFEN 3	4	2
GAOFEN 3-02	4	2
GAOFEN 3-03	3	2

**Table 4:** Simulation results of four algorithms under GCS and GTS methods.

Strategy	Algorithm	Profit of Belarus	Profit of Gabon
GCS	VNTS	0.9644	0.4168
	TS	0.8872	0.3846
	SA	0.8933	0.3918
	GA	0.8639	0.3773
GTS	VNTS	0.7940	0.3292
	TS	0.6800	0.2921
	SA	0.7094	0.2949
	GA	0.6137	0.2586

## Data Availability

The data presented in this study are available on request from the corresponding author.
